# Enabling High Efficiency Nanoplasmonics with Novel Nanoantenna Architectures

**DOI:** 10.1038/srep17562

**Published:** 2015-12-01

**Authors:** Moshik Cohen, Reuven Shavit, Zeev Zalevsky

**Affiliations:** 1 Faculty of Engineering, Bar-Ilan University, Ramat-Gan 52900, Israel; 2 Department of Electrical and Computer Engineering, Ben-Gurion University of the Negev, Beer-Sheva 84105, Israel; 3 Bar-Ilan Institute for Nanotechnology & Advanced Materials, Ramat-Gan 52900, Israel

## Abstract

Surface plasmon polaritons (SPPs) are propagating excitations that arise from coupling of light with collective electron oscillations. Characterized by high field intensity and nanometric dimensions, SPPs fashion rapid expansion of interest from fundamental and applicative perspectives. However, high metallic losses at optical frequencies still make nanoplasmonics impractical when high absolute efficiency is paramount, with major challenge is efficient plasmon generation in deep nanoscale. Here we introduce the *Plantenna*, the first reported nanodevice with the potential of addressing these limitations utilizing novel plasmonic architecture. The Plantenna has simple 2D structure, ultracompact dimensions and is fabricated on Silicon chip for future CMOS integration. We design the Plantenna to feed channel (20 nm × 20 nm) nanoplasmonic waveguides, achieving 52% coupling efficiency with Plantenna dimensions of λ^3^/17,000. We theoretically and experimentally show that the Plantenna enormously outperforms dipole couplers, achieving 28 dB higher efficiency with broad polarization diversity and huge local field enhancement. Our findings confirm the Plantenna as enabling device for high efficiency plasmonic technologies such as quantum nanoplasmonics, molecular strong coupling and plasmon nanolasers.

The unprecedented capability of nanometallic (e.g. plasmonic) structures to enhance and confine light into deep nanometer scale holds promise for large variety of applications^1^. Recently introduced solar cells^2^,^3^,^4^, biomedical imaging^5^,^6^,^7^ and ultrafast data processing devices^8^,^9^,^10^ based on optical nano plasmons provide strong evidence for the prominence of plasmonic technologies. However, typical plasmonic materials are characterized by high losses at optical frequencies, mainly attributed to interband transitions. Valence electrons absorbing the energy from a photon shift into the conduction band, resulting in loss^11^. These losses, which can hardly be compensated with even the best gain materials, make plasmonics impractical for applications which require high efficiency, such as all-optical switching and frequency conversion^12^. Recently, high efficiency excitation of propagating SPPs was achieved using nanofocusing^13^,^14^,^15^,^16^,^17^ and micrometer scale optical antennas^18^, with conversion efficiency exceeding 50%. However, the diffraction limited size of these devices results with poor integration capabilities with CMOS electronics and low compatibility for applications like quantum nanoplasmonics and energy harvesting, which require both subdiffraction dimensions and high field enhancement^19^,^20^,^21^,^22^,^23^. On the other hand, subwavelength devices such as bowtie^24^,^25^,^26^ and dipole^27^,^28^,^29^ nanoantennas enable optical excitation of localized surface plasmons with high intensity. However these devices, which design is borrowed from the microwave regime, achieve poor excitation efficiency of highly confined (<λ^3^/100,000 mode volumes) SPPs^30^,^31^,^32^. Therefore, overcoming the efficiency limitations of nanoplasmonics require new nanometric devices with high overall coupling efficiency. Here we introduce the Plantenna, a novel photon-plasmon nano–transducer, potentially capable to overcome the overall efficiency limitations of optical nanoplasmonics. The Plantenna is based on a new nano-architecture, engineered for extreme light enhancement and confinement. It is comprised of three coupled nanometallic strips, designed to achieve plasmonic excitations at multiple coupled interfaces. This nano - architecture provides numerous degrees of freedom for optimal balance between field enhancement, confinement and insertion loss. We design the Plantenna to feed channel nanoplasmonic waveguides with channel dimensions of 20 nm × 20 nm, achieving up to 52% efficiency at λ_0_ = 633 nm with Plantenna dimensions smaller than λ^3^/17,000. Our results confirm The Plantenna potential of addressing the overall efficiency limitation of optical nanoplasmonics.

## Results

**Figure 1:**
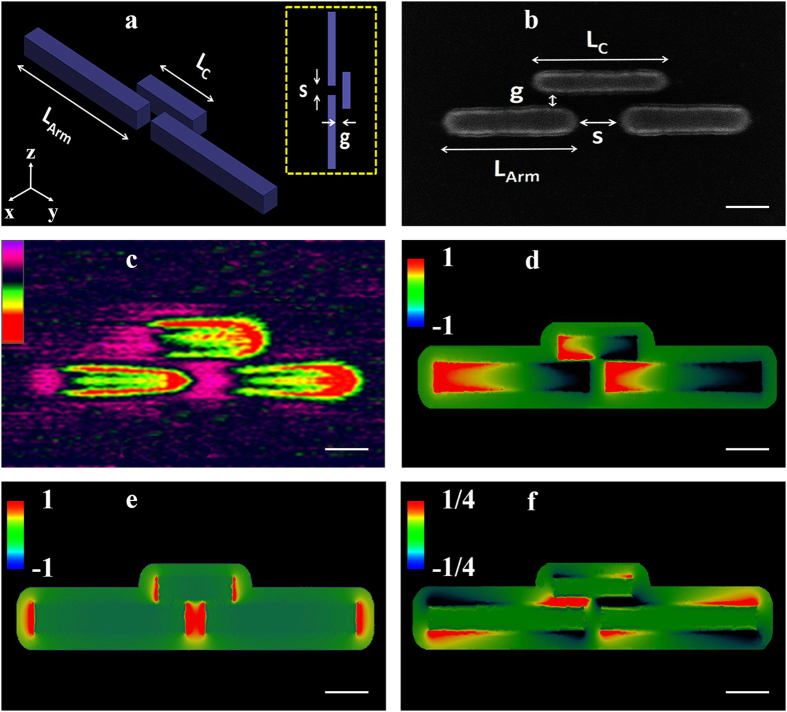
*Plantenna* architecture. (**a**) 3D configuration of the *Plantenna*, with the inset shows a top view in 2D. The two dipolar arms are aligned along the ***y*** axis, with the director spacing, g, is aligned with the ***x*** axis and the ***z***axis is normal to the substrate. (**b**) Ultra high resolution (0.7 nm) SEM image of a fabricated *Plantenna*. (**c**) KPFM under optical illumination characterization of the *Plantenna* KPFM. Signal scale bar: ±4.7 V. (**d**) Numerically calculated near-field showing Re(E_z_) = |E_z_|cos(ϕ_z_). (**e**) Numerically calculated near-field showing Re(E_x_). (**f)** Numerically calculated near-field showing Re(E_y_). Scale bar: 100 nm.

**Figure 2:**
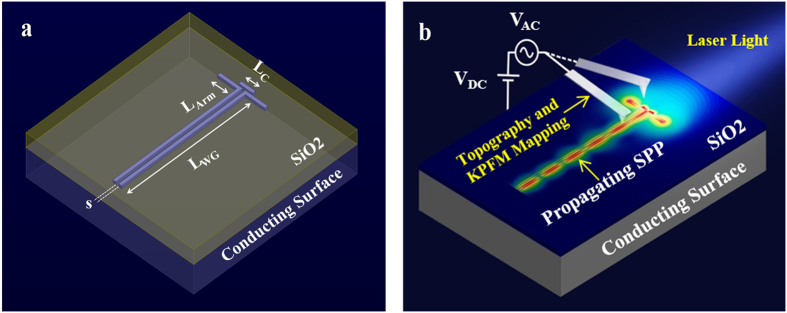
*Plantenna* coupler architecture and nanocharacterization setup. (**a**) 3D configuration of the *Plantenna*, coupled with channel nanoplasmonic waveguide (**b**) Experimental setup based on AFM and KPFM under optical illumination.

**Figure 3:**
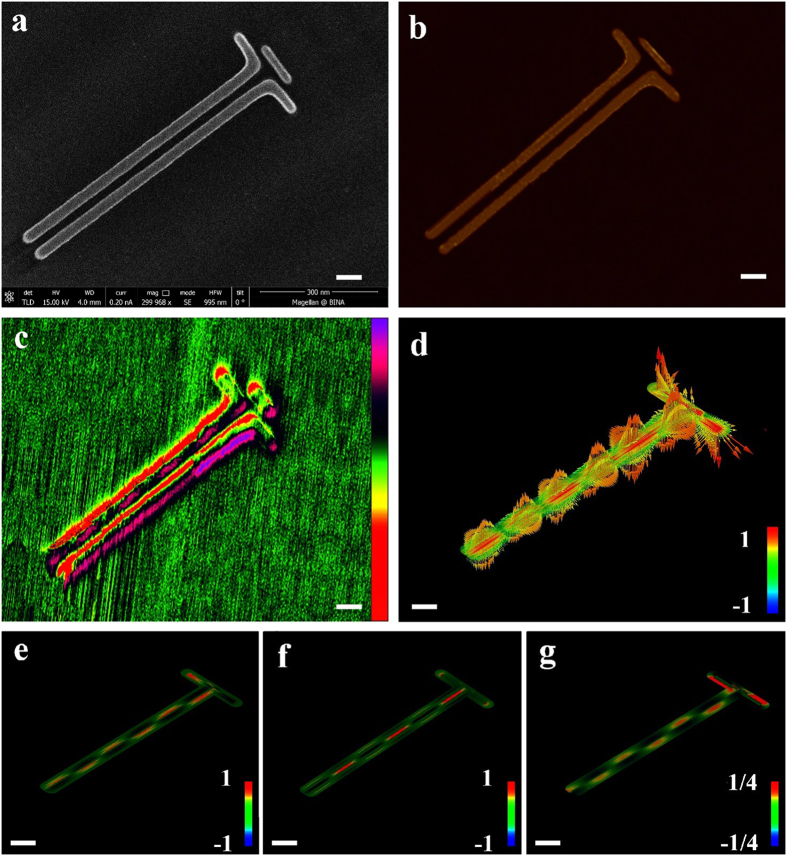
*Plantenna* coupler for channel nanoplasmonic waveguide. (**a**) High resolution SEM image of the fabricated device (**b**) 3D AFM image of the fabricated device (**c**) KPFM under optical illumination analysis of the device, KPFM Signal scale bar: ±4.7 V (**d)** Numerically calculated optical near field vector (**e**) Numerically calculated near-field showing Re(E_z_) = |E_z_|cos(ϕ_z_) (**f**) Numerically calculated near-field showing Re(E_x_) (**g**) Numerically calculated near-field showing Re(E_y_). Scale bar: 100 nm.

**Figure 4:**
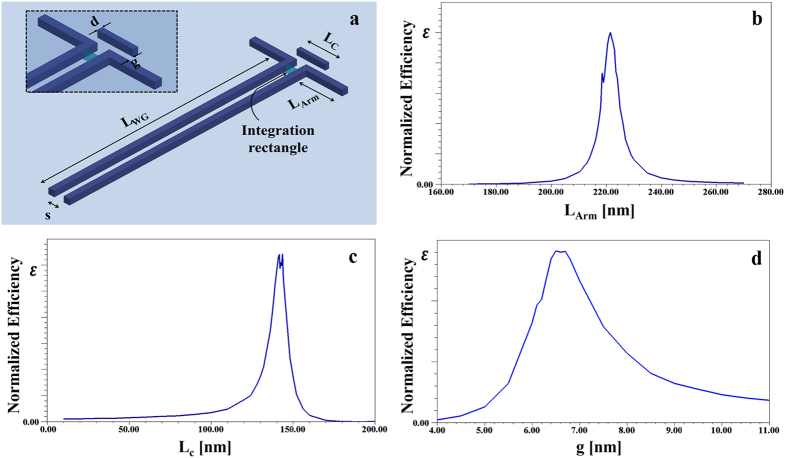
Numerical analysis of a *Plantenna* coupler for channel nanoplasmonic waveguide. (**a**) 3D configuration of the device, with the inset shows a zoom in on the Plantenna and channel cross section (**b**) Normalized efficiency vs *Plantenna* arm length (**c**) Normalized efficiency vs *Plantenna* director length (**d**) Normalized efficiency vs *Plantenna* director spacing.

**Figure 5:**
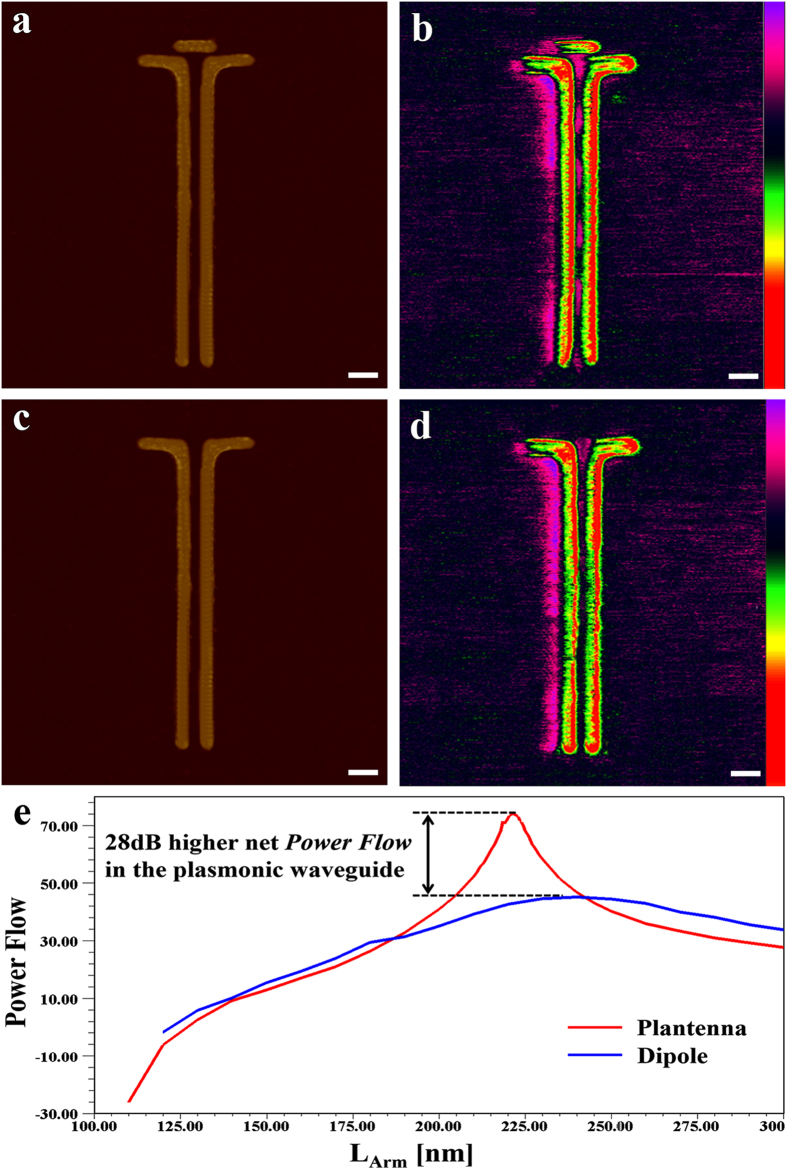
*Plantenna* vs dipole SPP coupler (**a**) 3D AFM image of a Plantenna coupled with channel nanoplasmonic waveguide (**b**) KPFM under optical illumination analysis of the device in (**a**). (**c**) 3D AFM image of a dipole coupled with identical waveguide (**d**) KPFM under optical illumination analysis of the device in (**c**). (**e)** comparison between the net plasmonic power flow inside the waveguide for Plantenna (red) and dipole (blue) coupler. Geometry scale bar: 100 nm. KPFM scale bar: ±4.7 V (**b**), ±0.4 V (**d**).

**Figure 6:**
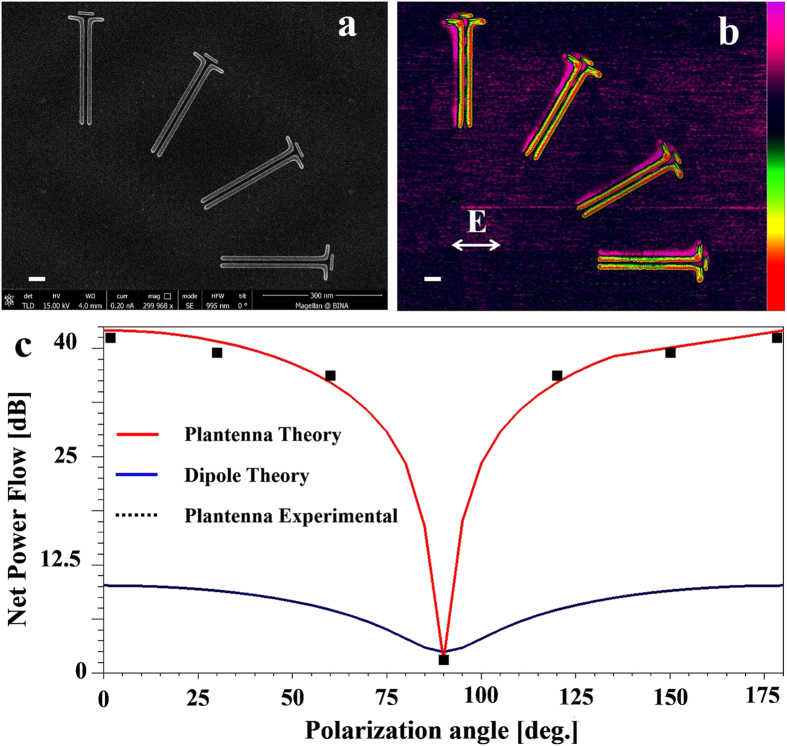
Polarization dependence efficiency of *Plantenna* coupler for channel nanoplasmonic waveguide. (**a**) High resolution SEM image of the fabricated devices (**b**) KPFM under optical illumination analysis of the devices, with rotation angles of of 
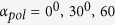
 and 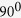, KPFM Signal scale bar: ±4.7 V (**c**) Net SPP power flow vs. polarization angle, theoretical and experimental analysis. Scale bar: 150 nm.

The fundamental principles inspire the Plantenna invention arise from the physics of coupled plasmonic nanoparticles^33^,^34^,^35^. At resonance, optically illuminated adjacent metallic particles exhibit enormous field confinement and enhancement, mainly due to coherent capacitive coupling. The Plantenna, schematically shown in Fig. 1a, is an engineered device comprised of three closely spaced metallic nanorods. Two identical nanorods of length L_Arm_ are separated by a nanoscopic gap (s = 10 nm–35 nm), in a dipole like arrangement. Additional nanorod, termed director, is placed at much closer proximity of only 6 nm (g = 6 nm) to the dipole. We use rigorous numerical simulations to optimize the structure, and provide experimental evidences that this arrangement leads to three coupled metallic interfaces with huge field enhancement and confinement. To achieve the resolution required for the Plantenna fabrication, we used electron beam lithography (EBL), ion beam sputtering (Ag, 20 nm) and liftoff with optimized beam doses. After liftoff, the resist is completely removed, allowing contact mode optical characterization in the near field. We fabricated devices of standalone Plantennas and integrated Plantenna and channel nanoplasmonic waveguides on standard Si substrate, demonstrating CMOS compatibility. Figure 1b shows a high-resolution scanning electron microscopy (HR-SEM) image of a fabricated Plantenna, recorded at beam current of 0.4 nA and low accelerating voltage of 5 kV, for sub-nanometer imaging resolution. Plantenna with demotions of L_Arm_ = 140 nm, L_C_ = 120 nm, s = 30 nm and g = 6 nm was fabricated successfully and repeatedly. Moreover, as shown in Fig. 1b, the nano-arms and director can be fabricated with different widths, providing an important degree of freedom for the Plantenna geometry optimization. We use Kelvin probe force microscopy (KPFM) under laser illumination for optical near field characterization. When a scanning probe tip is electrically connected to a conductive sample, a contact potential difference (CPD) will arise due to the different workfunctions of the tip and the sample. KPFM, a variant of atomic force microscopy (AFM), measures local variations in this CPD by applying a voltage between the sample and the oscillating AFM tip so that the electric field caused by the CPD and the resulting force on the tip are compensated. For a certain tip position, the compensating voltage represents the local contact potential difference (LCPD), which we recently introduced as a powerful tool for high resolution imaging of SPPs^8^,^36^,^37^,^38^. Here, we first acquire the topography of a single line in tapping mode and then retrace this topography over the same line at a set lift height from the surface to measure CPD. High-resolution KPFM images were recorded while the devices are illuminated by a He-Ne laser at wavelength of 633nm. To avoid coupling effects between the conducting Si and the metallic device we fabricated the structures on the insulating layer of a Si substrate^36^ (2 μm thick SiO2). Figure 1c shows high-resolution KPFM mapping of a Plantenna, recorded at s set lift height of 30 nm using high aspect ratio uncoated Si AFM tip with diameter of 2 nm. We observe huge plasmonic enhancement of the fields at the Plantenna gap, with deep nanoscale confinement. Moderate plasmonic enhancement is also observed at the uncoupled metal–air interfaces, a well-known property of metallic nanoparticles^8^,^36^,^38^. The Plantenna arms are inversely polarized, evidenced by opposite sign of the KPFM fields measured at the Plantenna edges. Inverse polarization pattern appears also at the director ends. This behavior is reproduced by numerical calculation results, presented at the optical frequency of 474 THz (633 nm). The numerical results are obtained using High Frequency Structure Simulator (ANSYS HFSS V15^8^,^39^,^40^) based on the finite element method (FEM). Generally, the electric field is described by a 3D vector **E** = (E_x_, E_y_, E_z_), where each field component *E*_*i*_ is characterized by both magnitude |*E*_*i*_| and phase ϕ_i_^41^. Figure 1d shows the real part of the vertical near-field component Re(E_z_)  = |E_z_|cos(ϕ_z_) where |E_z_| is the near-field amplitude and ϕ_z_ the phase. The E_z_ field distribution appears very similar to the KPFM map (Fig. 1c), exhibiting identical spatial phase distribution. For E_x_, the amplitude image exhibits a completely different pattern, featuring a highly localized, intense fields at the Plantenna gap and ends with spatially constant phase^26^,^36^. Although the transverse field component E_y_ has much smaller field intensity, it may result with slight shift of the phase center of the KPFM signal with respect to the topography^36^. We conclude that the main contributions to the KPFM map arise from the normal (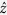) and transverse (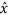) component of the electric field. The phase distribution and the field enhancement at the gap are governed by the normal and transversal field components, respectively. The huge field enhancement may lead to strong coupling when hybridized with active molecules, as currently being examined^42^. Next, we demonstrate the Plantenna supremacy in excitation of nanoscale SPPs. We compare the Plantenna with dipole nanoantennas, the most compact form of SPP couplers^8^,^14^,^36^,^43^. To this end we design, fabricate and characterize hybrid devices comprised of Plantenna and dipole couplers, integrated with channel nanoplasmonic waveguides, as illustrated in Fig. 2a. The devices are fabricated with Plantenna arm length of L_Arm_ = 220 nm, Director Length of L_C_ = 140 nm, channel cross section of 20 nm × 20 nm (s = 20 nm) and waveguide of 1.5 μm length. The director spacing is g = 7 nm and the director width is set to 12 nm. Figure 2b presents the experimental setup for AFM and KPFM measurements^8^,^36^,^38^. Figure 3a shows a HR SEM image of a fabricated hybrid device, comprised of a Plantenna integrated with channel nanoplasmonic waveguide, with 3D AFM topography of the device is shown in Fig. 3b. Figure 3c presents KPFM mapping under far field illumination with unfocused, linearly polarized He-Ne laser at wavelength of 633 nm covering the entire device. We observe dipolar-like modes on the Plantenna arms and director, with strong fields at the Plantenna ends and at the gap. The localized fields at the gap extend along the channel waveguide, periodically changing their polarity. The modal behavior of the propagating SPPs along the waveguide is observed in the KPFM image (Fig. 3c). Six periods of modes appear along the waveguide, represented by peaks of the KPFM signal, with the measured^8^,^36^,^37^,^38^ SPP wavelength is 245 nm. The shape of the waveguide, as well as the field decay into its metallic arms are very well captured. Strong localized fields are observed also on the exterior metallic interfaces of the waveguide, as predicted by the theory of SPPs^44^. The measured KPFM fields on the Plantenna ends are inversely polarized, with the field on the left edge is positive 4.7 V and the field on the right edge is negative −4.7 V, corresponds to local optical near field direction of 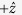
and 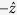, respectively (see Fig. 3d). The strong magenta line on the waveguide side appears due to an artifact originates from the scan direction, and therefore does not appear in the simulation results. Figure 3d presents the numerically calculated 3D electric near field vector, 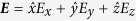, with each arrow represents the direction of the local electric near field vector. Similar to the KPFM map, high field enhancement is observed at the Plantenna ends and gap, with six periods of propagating SPPs along the waveguide. Additional insight into the optical field structure is obtained by analyzing the scalar near field components (E_x_, E_y_, E_z_), with the amplitude of each component is displayed on the sample surface (here, no arrows display is required). Figure 3e presents the real part of the vertical near-field component, Re(E_z_). We observe high intensity at the Plantenna ends and gap as well as periodical change in the field polarity, as previously reported for optical gap nanoantennas coupled to channel plasmon waveguides^14^,^36^. The horizontal component E_x_ exhibits a standing wave pattern with spatially constant phase along the Plantenna and waveguide^8^,^14^,^36^. Since E_x_ has significantly higher amplitude than E_z_, the phase behavior inside the waveguide is dominated by E_x_. This result explains why no phase difference between the fields inside the waveguide is observed in the KPFM image^36^ (Fig. 3c). The E_y_ component exhibits much lower field enhancement thus has a minor contribution to the overall field structure. The unique Plantenna architecture enables geometrical optimization for high efficiency photon-plasmon conversion. The coupling efficiency is defined as the ratio between the net SPP power flow and incident laser beam power -


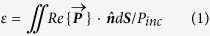


where 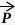 is the Poynting vector of the electromagnetic fields, 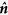 is a unit vector in the direction of wave propagation and 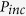 is incident laser beam power. The integration (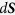) is performed on the waveguides channel cross section near (distance of 10nm) the Plantenna gap, avoiding effects of metallic propagation loss on the calculated efficiency. For larger distances, the efficiency decays exponentially, as governed by the propagation losses of SPPs inside the waveguide^8^. Figure 4a shows the analyzed configuration, with the waveguide channel dimensions are set for 20 nm × 20 nm (s = 20 nm) and the excitation is at 633 nm. Figure 4b,c present the SPP coupling efficiency, 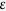, as a function of the arm (L_Arm_) and director (L_c_) length, respectively. Resonance behavior with narrow distribution is observed for both parameters, with resonance dimensions are L_Arm_ = 225nm and L_C_ = 140nm, achieving maximum efficiency of 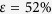. The most interesting design parameter is the director spacing, g. From Fig. 4d, we observe that only when the director is placed at a very close proximity to the Plantenna arms (e.g. 6.5 nm–7.5 nm) the high coupling efficiency achieved. For g > 8 nm the efficiency significantly decreases, asymptotically approaching the efficiency achieved by dipole nanoantenna couplers^30^. For g < 4 nm the efficiency sharply decreases, as we approach the limit of nonlocal regime for which additional quantum mechanical effects shall be considered^45^,^46^,^47^. Figure 5 presents a direct comparison between the Plantenna and dipole nanoantenna for SPP coupling in nanoplasmonic channel waveguides. Figure 5a shows an AFM image of a Plantenna integrated with nanoplasmonic waveguide (dimensions of L_Arm_ = 210 nm, L_C_ = 120 nm, s = 20 nm, g = 8 nm d = 20 nm and L_WG_ = 1.5 μm); with the KPFM analysis of the device is shown in Fig. 5b. A resonance, the Plantenna exhibits huge field enhancement which causes physical deformation and damage to the AFM cantilever. Therefore, the Plantenna in Fig. 5a is fabricated with a slightly off-resonance dimensions (L_Arm_ = 210 nm, g = 8 nm, compared with L_Arm_ = 225 nm, g = 6.5 nm required for maximum conversion efficiency, see Fig. 4). As shown in Fig. 5b, huge plasmonic field is excited at the Plantenna gap, subsequently extending into the channel and propagates along the waveguide. Small amount of radiation is observed outside the waveguide, as a slight portion of the propagating SPPs is recoupled to photons by the discontinuity at the waveguide open end. These results are compared to the case where an identical waveguide is connected to a resonant dipole nanoantenna, with AFM topography mapping of the device shown in Fig. 5c. The measured plasmonic intensity at the dipole gap shown in Fig. 5d is approximately 20 dB lower compared to the Plantenna case (Fig. 5b). The field at the dipole gap shows rapid decay inside the waveguide, with only the two first mode peaks of SPP propagation can be qualitatively observed. As a result, almost no radiation is observed (Fig. 5d) at the waveguide open end when coupling using dipole nanoantenna. Figure 5e shows a numerically calculated comparison of the net plasmonic power flow at the waveguide for SPP coupling using Plantenna (red) and dipole nanoantenna (blue) as a function of the arm length. Note that the coupling efficiency depends only in the net power flow (Eq.1). The dipole coupler exhibits broad behavior with moderate efficiency^18^,^30^,^43^ (power flow), peaking at L_Arm_ = 240 nm. However, the Plantenna coupler introduces a narrower response, with peak efficiency at L_Arm_ = 220 nm. The Plantenna achieves up to 28 dB higher efficiency compared with the conventional dipole. This enormous difference is almost entirely attributed to plasmonic effects, as the Plantenna area is only 20% larger than the dipole. Note that although optimized for resonant operation the diploe cannot exhibit similar efficiency pattern to the Plantenna, which has much more geometrical degrees of freedom for optimization. We use KPFM to analyze the effect of polarization angle on the Plantenna SPP coupling efficiency. In spherical coordinates system, we define the electric field vector as follows: 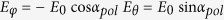; with 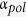 is the polarization angle and a fixed incident angle (**k** vector) of 45^0^. We characterize hybrid devices with similar dimensions to Fig. 5a, at polarization angles of 
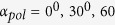
 and 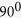. Figure 6a presents a HR SEM image of the characterized devices with the corresponding KPFM analysis is shown in Fig. 6b. We use a laser source without a polarizer; hence, the control of the polarization angle is achieved by fabricating devices with different angular orientation. Maximum coupling efficiency is achieved at 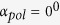, with moderate degradation until the region of 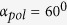. The efficiency drops when 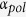 approaches 90^0^, similar to the case of dipole couplers. Figure 6c shows a detailed quantitative analysis of the polarization effect on coupling efficiency. The experimental results (black, dashed) are in good agreement with the numerical results (red), showing efficiency peak at 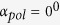 with sharp drop around 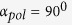
and symmetric behavior. For comparison, a numerical analysis of a dipole coupler is shown on the same graph (blue). Both Plantenna and dipole couplers exhibit efficiency minima at normal orientation (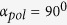). However, unlike the dipole, the efficiency of the Plantenna coupler rapidly increases, achieving 20dB better efficiency already at 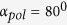. This huge different is attributed to the director orientation which enables resonance plasmonic behavior at wide spectrum of polarization angles.

In this work we introduce the Plantenna, a revolutionary nanoantenna exclusively designed for nanoplasmonics applications. The Plantenna utilizes a novel 2D plasmonic architecture to achieve the best-reported SPP coupling efficiency for a device smaller than λ^3^/15,000. Rigorous numerical calculations as well as optical nanocharacterization are used to design and investigate the Plantenna exciting near field structure. We show that the Plantenna enormously outperforms the conventional dipole coupler, achieving up to 28 dB higher SPP coupling efficiency with broad polarization diversity. We foresee the Plantenna to be the favorite plasmonic nanoantenna for tightly integrated plasmonic circuitry, all optical nano switching and strong light–matter interaction deep nanoscale.

## Methods

### AFM and KPFM measurements

All measurements were performed at room temperature and free ambient conditions (no vacuum), using Dimension Icon AFM system with NanoScope V controller (Bruker^®^). For both AFM and KPFM measurements, we used NanoWorld probes SSS-NCH, SuperSharpSilicon-Non-contact/Tapping™ mode-High resonance frequency; with typical diameter of 2 nm, resonance frequency of 320 kHz and spring constant of 42 N/m. typically, voltages of 2 V, ac capacitance frequencies of 880 MHz, lift heights of 30 nm−50 nm and line rates of 0.1 KHz were employed. To map the CPD of the sample, we apply both AC voltage (VAC) and a DC voltage (VDC) to the AFM tip. VAC generates oscillating electrical forces between the AFM tip and sample surface, and VDC nullifies the oscillating electrical forces that originated from CPD between tip and sample surface.

### Numerical simulations

The numerical results are obtained by using the software package ANSYS HFSS™ V15, the industry-standard simulation tool for 3D full-wave electromagnetic field simulation. HFSS solve Maxwell’s equations via the finite element method (FEM) using adaptive mesh refinement process for tailored accuracy requirements. The field’s solutions are calculated with the metallic (Ag) plasmonic structures being deposited on a homogenous SiO2 substrate. The nanoantenna is illuminated by optical sources at 474 THz (wavelength of 633 nm), which are modeled as focused Gaussian beams with 1μm characteristic diameter. The electric field is polarized in parallel with the dipole direction, as the wave vector **K** is perpendicular. A selectively dense meshing is assigned in the metallic and waveguiding regions, with maximum cell size of 1nm and 750,000 FEM tetrahedral cells. To provide maximum accuracy, the model is terminated as following: the interface with free space is bounded by perfectly matched layer (PML) absorbing boundary conditions (ABC), while the metallic and SiO2 termination are done via layered impedance (LI) ABC. The minimum number of adaptive meshing iterations was set to 12, with convergence condition of 1% maximum energy variance between adjacent iterations.

### Fabrication

SiO2/Si sample was spin-coated with poly (methyl methacrylate) (PMMA 950 A2) electron-beam resist providing thickness of 100 nm. The samples coated with PMMA were subsequently baked for 120 s on a hotplate at 180 C. The desired pattern was exposed in the PMMA layer using a CRESTEC CABLE-9000C high-resolution electron-beam lithography system using different doses to control line and gap width. Then the samples were developed for 90 sec using methyl isobutyl ketone (MIBK), and rinsed with IPA. The samples were subsequently exposed to Ar plasma to etch 10 nm in order to remove leftovers from the pattern, sputtered using BESTEC 2″ DC magnetron to deposit 3 nm Cr, and 17 nm Au, then immersed in 180 Khz ultrasonic bath with NMP for 3 h for resist liftoff.

## Additional Information

**How to cite this article**: Cohen, M. *et al.* Enabling High Efficiency Nanoplasmonics with Novel Nanoantenna Architectures. *Sci. Rep.* **5**, 17562; doi: 10.1038/srep17562 (2015).

## References

[b1] Brongersma M. L. & Shalaev V. M. The Case for Plasmonics. Science 328, 440–441 (2010). 20413483 10.1126/science.1186905

[b2] Ferry V. E., Sweatlock L. A., Pacifici D. & Atwater H. A. Plasmonic Nanostructure Design for Efficient Light Coupling into Solar Cells. Nano Lett. 8, 4391–4397 (2008). 19367883 10.1021/nl8022548

[b3] Green M. A. & Pillai S. Harnessing plasmonics for solar cells. Nat. Photonics 6, 130–132 (2012).

[b4] Tan H., Santbergen R., Smets A. H. M. & Zeman M. Plasmonic Light Trapping in Thin-film Silicon Solar Cells with Improved Self-Assembled Silver Nanoparticles. Nano Lett. 12, 4070–4076 (2012). 22738234 10.1021/nl301521z

[b5] Kumar S., Harrison N., Richards-Kortum R. & Sokolov K. Plasmonic Nanosensors for Imaging Intracellular Biomarkers in Live Cells. Nano Lett. 7, 1338–1343 (2007). 17439187 10.1021/nl070365i

[b6] Kawata S., Inouye Y. & Verma P. Plasmonics for near-field nano-imaging and superlensing. Nat. Photonics 3, 388–394 (2009).

[b7] Jun Y. *et al.* Continuous imaging of plasmon rulers in live cells reveals early-stage caspase-3 activation at the single-molecule level. Proc. Natl. Acad. Sci. USA 106, 17735–17740 (2009). 19805121 10.1073/pnas.0907367106 PMC2764940

[b8] Cohen M., Zalevsky Z. & Shavit R. Towards Integrated Nanoplasmonic Logic Circuitry. Nanoscale (2013). 10.1039/C3NR00830D. 23661298

[b9] Wei H., Wang Z., Tian X., Käll M. & Xu H. Cascaded logic gates in nanophotonic plasmon networks. Nat. Commun. 2, 387 (2011). 21750541 10.1038/ncomms1388 PMC3144585

[b10] Fu Y. *et al.* All-Optical Logic Gates Based on Nanoscale Plasmonic Slot Waveguides. Nano Lett. 12, 5784–5790 (2012). 23116455 10.1021/nl303095s

[b11] West P. r. *et al.* Searching for better plasmonic materials. Laser Photonics Rev. 4, 795–808 (2010).

[b12] Pile D. View from… SPP6: New directions in plasmonics. Nat. Photonics 7, 594–596 (2013).

[b13] Choo H. *et al.* Nanofocusing in a metal-insulator-metal gap plasmon waveguide with a three-dimensional linear taper. Nat. Photonics 6, 838–844 (2012).

[b14] Schnell M. *et al.* Nanofocusing of mid-infrared energy with tapered transmission lines. Nat. Photonics 5, 283–287 (2011).

[b15] Sorger V. J. *et al.* Experimental demonstration of low-loss optical waveguiding at deep sub-wavelength scales. Nat. Commun. 2, 331 (2011).

[b16] Pile D. F. P. & Gramotnev D. K. Adiabatic and nonadiabatic nanofocusing of plasmons by tapered gap plasmon waveguides. Appl. Phys. Lett. 89, 041111 (2006).

[b17] Verhagen E., Polman A. & Kuipers L. (Kobus). Nanofocusing in laterally tapered plasmonic waveguides. Opt. Express 16, 45–57 (2008). 18521131 10.1364/oe.16.000045

[b18] Kriesch A. *et al.* Functional Plasmonic Nanocircuits with Low Insertion and Propagation Losses. Nano Lett. 13, 4539–4545 (2013). 23962146 10.1021/nl402580c

[b19] Atwater H. A. & Polman A. Plasmonics for improved photovoltaic devices. Nat. Mater. 9, 205–213 (2010). 20168344 10.1038/nmat2629

[b20] Tame M. S. *et al.* Quantum plasmonics. Nat. Phys. 9, 329–340 (2013).

[b21] Pillai S., Catchpole K. R., Trupke T. & Green M. A. Surface plasmon enhanced silicon solar cells. J. Appl. Phys. 101, 093105–093105–8 (2007).

[b22] Derkacs D., Lim S. H., Matheu P., Mar W. & Yu E. T. Improved performance of amorphous silicon solar cells via scattering from surface plasmon polaritons in nearby metallic nanoparticles. Appl. Phys. Lett. 89, 093103 (2006).

[b23] Curto A. G. *et al.* Unidirectional Emission of a Quantum Dot Coupled to a Nanoantenna. Science 329, 930–933 (2010). 20724630 10.1126/science.1191922

[b24] Kinkhabwala A. *et al.* Large single-molecule fluorescence enhancements produced by a bowtie nanoantenna. Nat. Photonics 3, 654–657 (2009).

[b25] Liu N., Tang M. L., Hentschel M., Giessen H. & Alivisatos A. P. Nanoantenna-enhanced gas sensing in a single tailored nanofocus. Nat. Mater. 10, 631–636 (2011). 21572410 10.1038/nmat3029

[b26] Schnell M., Garcia-Etxarri A., Alkorta J., Aizpurua J. & Hillenbrand R. Phase-Resolved Mapping of the Near-Field Vector and Polarization State in Nanoscale Antenna Gaps. Nano Lett. 10, 3524–3528 (2010). 20701270 10.1021/nl101693a

[b27] Seok T. J. *et al.* Radiation Engineering of Optical Antennas for Maximum Field Enhancement. Nano Lett. 11, 2606–2610 (2011). 21648393 10.1021/nl2010862

[b28] Novotny L. & van Hulst N. Antennas for light. Nat. Photonics 5, 83–90 (2011).

[b29] Schnell M. *et al.* Controlling the near-field oscillations of loaded plasmonic nanoantennas. Nat. Photonics 3, 287–291 (2009).

[b30] Wen J., Romanov S. & Peschel U. Excitation of plasmonic gap waveguides by nanoantennas. Opt. Express 17, 5925–5932 (2009). 19365411 10.1364/oe.17.005925

[b31] Huang J.-S., Feichtner T., Biagioni P. & Hecht B. Impedance Matching and Emission Properties of Nanoantennas in an Optical Nanocircuit. Nano Lett. 9, 1897–1902 (2009). 19338279 10.1021/nl803902t

[b32] Alù A. & Engheta N. Wireless at the Nanoscale: Optical Interconnects using Matched Nanoantennas. Phys. Rev. Lett. 104, 213902 (2010). 20867100 10.1103/PhysRevLett.104.213902

[b33] Aizpurua J. *et al.* Optical properties of coupled metallic nanorods for field-enhanced spectroscopy. Phys. Rev. B 71, 235420 (2005).

[b34] Papavassiliou G. C. Optical properties of small inorganic and organic metal particles. Prog. Solid State Chem. 12, 185–271 (1979).

[b35] Hallock A. J., Redmond P. L. & Brus L. E. Optical forces between metallic particles. Proc. Natl. Acad. Sci. USA 102, 1280–1284 (2005). 15647359 10.1073/pnas.0408604101 PMC547848

[b36] Cohen M., Shavit R. & Zalevsky Z. Observing Optical Plasmons on a Single Nanometer Scale. Sci. Rep. 4, (2014). 10.1038/srep04096. PMC3930893 24556874

[b37] Cohen M., Shavit R., Zalevsky Z. & Abulafia Y. Nanoplasmonic Phased Array Superlens with Extended Depth of Focus. In *CLEO: 2014* JTu4A.135 (Optical Society of America, 2014). 10.1364/CLEO_AT.2014.JTu4A.135.

[b38] Cohen M., Shavit R. & Zalevsky Z. In Planar Waveguides and other Confined Geometries (ed. Marowsky G.) 45–66 (Springer: New York,, 2015) (Date of access: 01/01/2015)″. at http://link.springer.com/chapter/10.1007/978-1-4939-1179-0_3.

[b39] Vicarelli L. *et al.* Graphene field-effect transistors as room-temperature terahertz detectors. Nat. Mater. 11, 865–871 (2012). 22961203 10.1038/nmat3417

[b40] Murray W. A., Suckling J. R. & Barnes W. L. Overlayers on Silver Nanotriangles: Field Confinement and Spectral Position of Localized Surface Plasmon Resonances. Nano Lett. 6, 1772–1777 (2006). 16895372 10.1021/nl060812e

[b41] Gersen H., Novotny L., Kuipers L. & van Hulst N. F. On the concept of imaging nanoscale vector fields. Nat. Photonics 1, 242–242 (2007).

[b42] Carmeli I. *et al.* Spatial modulation of light transmission through a single microcavity by coupling of photosynthetic complex excitations to surface plasmons. Nat. Commun. 6, (2015). 10.1038/ncomms8334. 26055942

[b43] Andryieuski A., Malureanu R., Biagi G., Holmgaard T. & Lavrinenko A. Compact dipole nanoantenna coupler to plasmonic slot waveguide. Opt. Lett. 37, 1124–1126 (2012). 22446246 10.1364/OL.37.001124

[b44] Pitarke J. M., Silkin V. M., Chulkov E. V. & Echenique P. M. Theory of surface plasmons and surface-plasmon polaritons. Rep. Prog. Phys. 70, 1 (2007).

[b45] Esteban R., Borisov A. G., Nordlander P. & Aizpurua J. Bridging quantum and classical plasmonics with a quantum-corrected model. Nat. Commun. 3, 825 (2012). 22569369 10.1038/ncomms1806

[b46] Savage K. J. *et al.* Revealing the quantum regime in tunnelling plasmonics. Nature 491, 574–577 (2012). 23135399 10.1038/nature11653

[b47] Scholl J. A., García-Etxarri A., Koh A. L. & Dionne J. A. Observation of Quantum Tunneling between Two Plasmonic Nanoparticles. Nano Lett. 13, 564–569 (2013). 23245286 10.1021/nl304078v

